# Inference for Gaussian Processes with Matérn Covariogram on Compact Riemannian Manifolds

**Published:** 2023-03

**Authors:** Didong Li, Wenpin Tang, Sudipto Banerjee

**Affiliations:** Department of Biostatistics, University of North Carolina at Chapel Hill, Chapel Hill, NC 27599, USA; Department of Industrial Engineering and Operations Research, Columbia University, New York, NY 10027, USA; Department of Biostatistics, University of California, Los Angeles, Los Angeles, CA 90095 USA

**Keywords:** Equivalence of Gaussian measures, Identifiability and consistency, Laplace-Beltrami operator, Microergodic parameters

## Abstract

Gaussian processes are widely employed as versatile modelling and predictive tools in spatial statistics, functional data analysis, computer modelling and diverse applications of machine learning. They have been widely studied over Euclidean spaces, where they are specified using covariance functions or covariograms for modelling complex dependencies. There is a growing literature on Gaussian processes over Riemannian manifolds in order to develop richer and more flexible inferential frameworks for non-Euclidean data. While numerical approximations through graph representations have been well studied for the Matérn covariogram and heat kernel, the behaviour of asymptotic inference on the parameters of the covariogram has received relatively scant attention. We focus on asymptotic behaviour for Gaussian processes constructed over compact Riemannian manifolds. Building upon a recently introduced Matérn covariogram on a compact Riemannian manifold, we employ formal notions and conditions for the equivalence of two Matérn Gaussian random measures on compact manifolds to derive the parameter that is identifiable, also known as the microergodic parameter, and formally establish the consistency of the maximum likelihood estimate and the asymptotic optimality of the best linear unbiased predictor. The circle is studied as a specific example of compact Riemannian manifolds with numerical experiments to illustrate and corroborate the theory.

## 1. Introduction

Gaussian processes are pervasive in spatial statistics, functional data analysis, computer modelling and machine learning applications because of the flexibility and richness they allow in modelling complex dependencies (Rasmussen and Williams, 2006; Stein, 1999; Gelfand et al., 2010; Cressie and Wikle, 2011; Banerjee et al., 2015). For example, in spatial statistics Gaussian processes are widely used to model spatial dependencies in geostatistical models and perform spatial prediction or interpolation (“kriging”) (Matheron, 1963). In non-parametric regression models Gaussian processes are used to model unknown functions and, specifically in Bayesian contexts, act as priors over functions (Ghosal and van der Vaart, 2017). A typical modelling framework assumes y(x)=μ(x)+Z(x)+ϵ(x) for inputs x (e.g., spatial coordinates; functional inputs) over a domain 𝒟, where y(x) is a dependent variable of interest, μ(x) is a mean function, Z(x) is a zero-mean Gaussian process and ϵ(x) is a noise process^1^. These frameworks can also be adapted to deal with discrete outcomes and applied to classification problems (Neal, 1999). Gaussian processes are also being increasingly employed in deep learning and reinforcement learning (Damianou and Lawrence, 2013; Deisenroth et al., 2013). The current manuscript focuses upon inferential properties of Z(x) when 𝒟 is not necessarily Euclidean but a compact Riemannian manifold.

A Gaussian process is determined by its covariogram, also known as the covariance function. In Euclidean space, the Matérn covariogram (Matérn, 1986) is especially popular in spatial statistics and machine learning (see, e.g., Stein, 1999, for an extensive discussion on the theoretical properties of the Matérn covariogram). A key attraction of the Matérn covariogram is the availability of a smoothness parameter for the process. Several simpler covariograms, such as the exponential, arise as special cases of the Matérn.

This article is motivated by the emergence of non-Euclidean data, especially manifold data, in a variety of scientific fields over the last decade. As a consequence, inference for Gaussian processes on manifolds have been attracting attention in spatial statistics and machine learning in settings where the data generating process is more appropriately mod-elled over non-Euclidean spaces. Taking climate science as an example, geographic data involving geopotential height, temperature and humidity are measured at global scales and are more appropriately treated as (partial) realisations of a spatial process over a sphere (see, e.g., Banerjee, 2005; Jun and Stein, 2008; Jeong and Jun, 2015a). Data arising over domains with irregular shapes or examples in biomedical imaging where the domain is a three-dimensional shape of an organ comprise other examples where inference for Gaussian processes over manifolds will be relevant (see, e.g., Gao et al., 2019, and references therein). Motivated by isotropic covariograms in Euclidean space, it is natural to replace Euclidean distance by an appropriate geodesic distance to define a “Matérn” covariogram on Riemannian manifolds. However, this formal generalisation is not valid for the squared exponential covariogram, or Matérn with ν=∞ (Feragen et al., 2015), unless the manifold is flat. For Matérn with ν∈(1/2,∞), this naive generalisation is not even valid on the sphere (Gneiting, 2013). Recently, valid covariograms for smooth Gaussian processes on general Riemannian manifolds have been constructed based upon heat equations, Brownian motion and diffusion models on manifolds (Castillo et al., 2014; Niu et al., 2019; Dunson et al., 2020). However, these covariograms lack flexibility, especially in terms of modelling smoothness.

Whittle (1963) proposed a new representation of GP by stochastic partial differential equations. Following this path, Lindgren et al. (2011) introduced a “Matérn” family on generic compact Riemannian manifolds with three parameters involved in the covariogram. Since such Matérn covariograms involve the spectrum of the Laplace-Beltrami operator, a numerical approximation to the covariogram is needed for most nontrivial manifolds. There is a rich literature focusing on approximations to the covariogram using tools from harmonic analysis, graph Laplacians, and stochastic partial differential equations (Sanz-Alonso and Yang, 2022a b). However, the study of statistical inference for the parameters in the Matérn covariogram remains relatively sparse.

In Euclidean domains $R^{d}$ with d≤3, while not all parameters in the Matérn covariogram are consistently estimable within the paradigm of “fixed-domain” or “in-fill” asymptotic inference (see, e.g. Stein, 1999; Zhang, 2004), certain parameters, customarily referred to as microergodic parameters, which can identify Gaussian processes specified by Matérn covariograms are consistently estimable (see Section 2). Furthermore, the maximum likelihood estimator of the spatial variance under any misspecified decay parameter is consistently and asymptotically normally distributed (Du et al., 2009; Kaufman et al., 2008; Wang and Loh, 2011), while predictive inference is also asymptotically optimal using maximum likelihood estimators (Kaufman and Shaby, 2013). Recently, Bevilacqua et al. (2019) and Ma and Bhadra (2022) considered more general classes of covariance functions outside of the Matérn family and studied the consistency and asymptotic normality of the maximum likelihood estimator for the corresponding microergodic parameters.

Our current contribution develops asymptotic inference for a flexible and rich Matérn-type covariogram on compact Riemannian manifolds. We review the Matérn covariogram (Section 3.1) on general compact Riemannian manifolds from the perspective of stochastic partial differential equations with reasonably tractable covariograms and spectral densities (Borovitskiy et al., 2020). Our specific results emanate from a sufficient and necessary condition for the equivalence of two Gaussian random measures on compact Riemannian manifolds with Matérn or squared exponential covariograms (Section 3.2). We subsequently establish (Section 3.3) that for Gaussian measures with Matérn covariograms the smoothness parameter is identifiable, while the spatial variance and decay parameters are not identifiable when d≤3, where d is the dimension of the manifold. For d≥4, all three parameters are identifiable. For squared exponential covariograms on manifolds with arbitrary dimension, we show that both parameters are identifiable. Again, this problem is still open in Euclidean spaces. For Matérn covariograms on manifolds with d≤3, we formally establish that the maximum likelihood estimate of the spatial variance with a misspecified decay parameter is still consistent. Next, we turn to predictive inference (Section 3.4) and show that for any misspecified decay parameter in the Matérn covariogram, the best linear unbiased predictor derived from the maximum likelihood estimate is asymptotically optimal. Finally, for spheres with dimension less than 4, we explicitly study the Matérn covariogram, the microergodic parameter, the consistency of the maximum likelihood estimate and the optimality of the best linear unbiased predictor (Section 4). Proofs and mathematical details surrounding our main results are provided in the Appendix.

## 2. Gaussian Processes in Euclidean spaces

Let $Z=\left\{Z(x):x\in ℳ\subset R^{d}\right\}$ be a zero-mean Gaussian process on a bounded domain ℳ. The process Z(⋅) is characterised by its covariogram k(x,y)=E(Z(x)Z(y)),x,y∈ℳ so that for any finite collection of points, say $x_{1},\cdots ,x_{n}\in ℳ$, we have ${\left(Z\left(x_{1}\right),\cdots ,Z\left(x_{n}\right)\right)}^{T}\sim \;𝒩(0,\Sigma )$, where Σ is the n×n covariance matrix with (i,j)-th entry $\Sigma_{ij}=k\left(x_{i},x_{j}\right)$. The Matérn process is a zero-mean stationary Gaussian process specified by the covariogram^2,^

(1) $$k(x,y)=\frac{\sigma^{2}(\alpha ∥x-y∥{)}^{\nu }}{\Gamma (\nu )2^{\nu -1}}K_{\nu }(\alpha ∥x-y∥),\;x,y\in ℳ\subset R^{d},$$

where $\sigma^{2}>0$ is called the partial sill or spatial variance, α>0 is the scale or decay parameter, ν>0 is a smoothness parameter, Γ(⋅) is the Gamma function, and $K_{\nu }(\cdot )$ is the modified Bessel function of the second kind of order ν (Abramowitz and Stegun, 1965, Section 10). The Matérn covariogram in (1) is isotropic and its spectral density (also known as the Hankel-Fourier transform, Genton (2002)) is given by

$$f(u)=\frac{\sigma^{2}\alpha^{2\nu }}{\pi^{d/2}{\left(\alpha^{2}+u^{2}\right)}^{\nu +d/2}},\;u\geq 0.$$

### 2.1. Identifiability

Let $P_{0}$ and $P_{1}$ be Gaussian measures corresponding to Matérn parameters $\left\{\sigma_{0}^{2},\alpha_{0},\nu \right\}$ and $\left\{\sigma_{1}^{2},\alpha_{1},\nu \right\}$, respectively. Two measures are said to be equivalent, denoted by $P_{0}\equiv P_{1}$, if they are absolutely continuous with respect to each other. Two equivalent measures cannot be distinguished no matter how dense the observations are. Zhang (2004) showed that when $d<4,P_{0}$ is equivalent to $P_{1}$ if and only if $\sigma_{0}^{2}\alpha_{0}^{2\nu }=\sigma_{1}^{2}\alpha_{1}^{2\nu }$. Hence, $\sigma^{2}$ and α do not admit asymptotically consistent estimators, while $\sigma^{2}\alpha^{2\nu }$, also known as a *microergodic parameter*, is consistently estimable. For d>4, Anderes (2010) proved that both $\sigma^{2}$ and α are consistently estimable. The case for d=4 remains unresolved. The integral test offers a sufficient (but not necessary) condition on the spectral densities to determine whether two measures are equivalent. While unidentifiable parameters are never consistently estimable, identifiable parameters may be consistently estimable. However, deriving an explicit construction for such a consistent estimator is often challenging and is beyond the scope of the current manuscript; we identify this as an area of future research.

### 2.2. Parameter estimation

In practice, the maximum likelihood estimate is customarily used to estimate unknown parameters in the covariogram. Let $L_{n}\left(\sigma^{2},\alpha \right)$ be the likelihood function:

(2) $$L_{n}\left(\sigma^{2},\alpha \right)={\left(2\pi \sigma^{2}\right)}^{-n/2}\text{det}\;{\left(\Gamma_{n}\left(\alpha \right)\right)}^{-\frac{1}{2}}\text{exp}\;\left\{-\frac{1}{2\sigma^{2}}Z_{n}^{T}\Gamma_{n}(\alpha {)}^{-1}Z_{n}\right\},$$

where $Z_{n}={\left(Z\left(x_{1}\right),\cdots ,Z\left(x_{n}\right)\right)}^{T}$ and ${\left(\Gamma_{n}(\alpha )\right)}_{i,j}=\frac{{\left(\alpha ∥x_{i}-x_{j}∥\right)}^{\nu }}{\Gamma (\nu )2^{\nu -1}}K_{\nu }\left(\alpha ∥x_{i}-x_{j}∥\right)$ is independent of $\sigma^{2}$. Given α, the maximum likelihood estimation of $\sigma^{2}$ is given by (Stein, 1999)

$${\hat{\sigma }}^{2}=\frac{Z_{n}^{T}\Gamma_{n}(\alpha {)}^{-1}Z_{n}}{n}.$$

Let $\left\{\sigma_{0}^{2},\alpha_{0}\right\}$ be the data generating parameters with observations $Z\left(x_{1}\right),\cdots ,Z\left(x_{n}\right)$. For any misspecified $\alpha_{1}$, if ${\hat{\sigma }}_{1,n}^{2}$ is the maximum likelihood estimation of $L_{n}\left(\sigma^{2},\alpha_{1}\right)$, then ${\hat{\sigma }}_{1,n}^{2}\alpha_{1}^{2\nu }\to \sigma_{0}^{2}\alpha_{0}^{2\nu }$ as n→∞ with probability 1 under $P_{0}$ when $\cup_{n=1}^{\infty }\left\{x_{n}\right\}$ is bounded and infinite (Zhang, 2004; Kaufman et al., 2008). Moreover, $\sqrt{n}\left(\frac{{\hat{\sigma }}_{1,n}^{2}\alpha_{1}^{2\nu }}{\sigma_{0}^{2}\alpha_{0}^{2\nu }}-1\right)\to 𝒩(0,2)$ as n→∞ (Du et al., 2009; Wang and Loh, 2011; Kaufman and Shaby, 2013). As a result, even if we do not know the true parameters $\left\{\alpha_{0},\sigma_{0}^{2}\right\}$, we can choose an arbitrary, possibly misspecified, decay parameter $\alpha_{1}$ and find the maximum likelihood estimate of the spatial variance ${\hat{\sigma }}_{1,n}^{2}$. The resulting Gaussian measure is asymptotically equivalent to the Gaussian measure corresponding to the true parameter.

### 2.3. Prediction and kriging

Gaussian processes are widely deployed in spatial or nonparametric regression models to carry out model-based predictive inference. Given a new location $x_{0}$, the best linear unbiased predictor (BLUP) for $Z_{0}=Z\left(x_{0}\right)$ is given by

$${\hat{Z}}_{n}(\alpha )=\gamma_{n}(\alpha {)}^{T}\Gamma_{n}(\alpha {)}^{-1}Z_{n},$$

where ${\left(\gamma_{n}(\alpha )\right)}_{i}=\frac{{\left(\alpha ∥x_{0}-x_{i}∥\right)}^{\nu }}{\Gamma (\nu )2^{\nu -1}}K_{\nu }\left(\alpha ∥x_{0}-x_{i}∥\right)$. Then

$$\frac{E_{\sigma_{0}^{2},\alpha_{0}}{\left({\hat{Z}}_{n}\left(\alpha_{1}\right)-Z_{0}\right)}^{2}}{E_{\sigma_{0}^{2},\alpha_{0}}{\left({\hat{Z}}_{n}\left(\alpha_{0}\right)-Z_{0}\right)}^{2}}\overset{n\to \infty }{⟶}1,\frac{E_{{\hat{\sigma }}_{1,n}^{2},\alpha_{1}}{\left({\hat{Z}}_{n}\left(\alpha_{1}\right)-Z_{0}\right)}^{2}}{E_{\sigma_{0}^{2},\alpha_{0}}{\left({\hat{Z}}_{n}\left(\alpha_{1}\right)-Z_{0}\right)}^{2}}\overset{n\to \infty }{⟶}1,$$

where E is the expectation with respect to the measure characterised by the parameter or spectral density (see Section 3) in the subscript. As a result, any misspecified α still yields an asymptotically optimal BLUP as long as $\sigma^{2}$ is replaced by its maximum likelihood estimate (Stein, 1993; Kaufman and Shaby, 2013). In the current manuscript, we develop parallel results for the d dimensional compact Riemannian manifold ℳ.

## 3. Gaussian processes on compact Riemannian manifold

Henceforth, we assume that our domain of interest is a d-dimensional compact Riemannian manifold ℳ equipped with a Riemannian metric g. We denote the Laplace–Beltrami operator on ℳ by–$\Delta_{g}$ with eigenvalues $\lambda_{n}$ and eigenfunctions $f_{n}$, the volume form by $dV_{g}$ and the volume of ℳ by $V_{ℳ}$ (see, e.g., Kobayashi and Nomizu, 1963; Lee, 2018; do Carmo, 1992, for further details on operators and spectral theory on Riemannian manifolds).

### 3.1. Matérn covariogram on compact Riemannian manifolds

On a Riemannian manifold, where the linear structure of $R^{d}$ is missing, the standard definition of the Matérn covariogram is no longer valid. A natural extension of the Matérn covariogram to manifolds will consider replacing the Euclidean norm ∥x-y∥ in (1) by the geodesic distance d(x,y). Unfortunately, this naive generalisation is not valid for ν=∞ (Feragen et al., 2015), unless the manifold is flat. If we restrict ourselves to spheres, Matérn with ν∈(1/2,∞) is still invalid (Gneiting, 2013). Instead, some Matérn-like covariograms including chordal, circular and Legendre Matérn covariograms and other families of covariograms have been studied (Jeong and Jun, 2015b; Porcu et al., 2016; Guinness and Fuentes, 2016; Guella et al., 2018; Clarke De la Cerda et al., 2018; Alegría et al., 2021). However, these covariograms are constructed specifically with respect to the geometry of the sphere and do not generalise to generic compact Riemannian manifolds.

Whittle (1963) showed that the Matérn covariogram in Euclidean space admits a representation through a stochastic partial differential equation involving white noise and the Laplace operator Δ. Lindgren et al. (2011) built on this stochastic partial differential equation approach to define the Matérn covariogram on manifolds involving the Laplace-Beltrami operator $\Delta_{g}$. This idea was further developed, both theoretically and practically, by several scholars (see, e.g., Bolin and Lindgren, 2011; Lang and Schwab, 2015; Herrmann et al., 2020; Borovitskiy et al., 2020, 2021, among others). We state the definition of the Matérn covariogram in the stochastic partial differential equation sense, which is a valid positive definite function for any ν on any compact Riemannian manifold ℳ.

**Definition 1** *Let* $f_{l}$ *be the orthonormal eigenfunctions of* -$\Delta_{g}$ *and* $\lambda_{l}\geq 0$ *be the corresponding eigenvalues in ascending order. The Matérn covariogram is defined by*

$$k\left(x,y\right)=\frac{\sigma^{2}}{C_{\nu ,\alpha }}\sum_{l=0}^{\infty }\;{\left(\alpha^{2}+\lambda_{l}\right)}^{-\nu -\frac{d}{2}}f_{l}\left(x\right)f_{l}\left(y\right),$$

*where* $C_{\nu ,\alpha }=\sum_{l=0}^{\infty }{\left(\alpha^{2}+\lambda_{l}\right)}^{-\nu -d/2}$ *is a constant such that the average variance is $\sigma^{2}=$* $\frac{1}{V_{ℳ}}\int_{ℳ}\;k(x,x)dV_{g}(x)$. *The corresponding spectral density is*

$$\rho \left(l\right)=\frac{\sigma^{2}}{C_{\nu ,\alpha }}{\left(\alpha^{2}+\lambda_{l}\right)}^{-\nu -\frac{d}{2}}.$$

Similarly, the squared exponential covariogram is

$$k\left(x,y\right)=\frac{\sigma^{2}}{C_{\infty ,\alpha }}\sum_{l=0}^{\infty }\;e^{-\frac{\lambda_{l}}{2\alpha^{2}}}f_{l}\left(x\right)f_{l}\left(y\right),$$

*where* $C_{\infty ,\alpha }=\sum_{l=0}^{\infty }e^{-\frac{1}{2\alpha^{2}}\lambda_{l}}$ *is a constant such that the average variance is* $\sigma^{2}=\frac{1}{V_{ℳ}}\int_{ℳ}\;k(x,x){dV}_{g}(x)$.

The corresponding spectral density is

$$\rho \left(n\right)=\frac{\sigma^{2}}{C_{\infty ,\alpha }}e^{-\frac{\lambda_{l}}{2\alpha^{2}}}.$$

**Remark 2** There are several commonly used parametric representations of the Matérn covariogram. In particular, this article adopts the same parametric representation as the one in Zhang (2004), but different from Borovitskiy et al. (2021).

If ℳ is a sphere, the covariograms defined above coincide with the Matérn-like covariograms on spheres provided by Guinness and Fuentes (2016) and Kirchner and Bolin (2022). As a result, we focus on a non-trivial generalisation to generic compact Riemannian manifolds. The relation between the three parameters $\left(\alpha ,\sigma^{2},\nu \right)$ in the above definition and the coefficients in the stochastic partial differential equation representation is not straightforward (see Lindgren et al., 2011, for details). Note that for any $\left(\alpha ,\sigma^{2},\nu \right)$, the covariogram shares the same eigenbasis with the Laplace-Beltrami operator $\Delta_{g}$. This property is not deemed restrictive for our ensuing development since we primarily focus on the Matérn and squared exponential covariograms. Furthermore, this property offers crucial analytic tractability for several results developed subsequently. Hence, we refer to the Matérn and squared exponential covariograms as in Definition 1 in the following sections.

### 3.2. Identifiability

In Euclidean domains, the integral test (Yadrenko, 1983; Stein, 1999) is a powerful tool to determine the equivalence of two Gaussian measures. However, such tests do not carry through to non-Euclidean domains as the spectrum on such manifolds is discrete. Alegría et al. (2021) studied the so called ℱ-family of covariograms on spheres and numerically deduced, without proof, the consistency of the maximum likelihood estimate of some parameters for this family. Arafat et al. (2018) derived the equivalence of Gaussian measures on spheres and derived microergodic parameters of some covariograms excluding the Matérn. All of the above results are built upon the Feldman-Hájek Theorem (Da Prato and Zabczyk, 2014), which is valid for any metric space and, hence, applicable to compact Riemannian manifolds. Here, we generalise the above results to a Gaussian process with Matérn and squared exponential covariograms on arbitrary compact Riemannian manifolds, also motivated by the Feldman-Hájek theorem. Therefore, we can still study the identifiability of these parameters by finding the microergodic parameters.

**Lemma 3** *Let* $P_{i}(i=1,\;2)$ *be mean zero Matérn/squared exponential Gaussian random measures with spectral densities* $\rho_{i}$. *Then*, $P_{1}\equiv P_{2}$ *if and only if*

$$\sum_{l}\;{\left|\frac{\rho_{2}(l)-\rho_{1}(l)}{\rho_{1}(l)}\right|}^{2}<\infty .$$

**Proof** See Appendix A. ■

From Definition 1, $\rho_{i}$ is strictly positive so the denominator is always non-zero. The series test is a sufficient and necessary condition. This is a significant enhancement over the integral test in Euclidean spaces, which offers only a sufficient condition. Its importance to us will become clear after Theorem 4. Subsequently, we consider microergodic parameters of Gaussian processes on a manifold with the Matérn covariogram. This is analogous to Theorem 2 in Zhang (2004) for compact Riemannian manifolds.

**Theorem 4** *Let* $P_{i},i=1,2$, *denote two Gaussian measures with the Matérn covariogram parametrized by* $\theta_{i}=\left\{\sigma_{i}^{2},\alpha_{i},\nu_{i}\right\}$. *Then the following results hold*.

- *If* d≤3, *then* $P_{1}\equiv P_{2}$ *if and only if* $\sigma_{1}^{2}/C_{\nu_{1},\alpha_{1}}=\sigma_{2}^{2}/C_{\nu_{2},\alpha_{2}},\nu_{1}=\nu_{2}$.
- *If* d≥4, *then* $P_{1}\equiv P_{2}$ *if and only if* $\sigma_{1}^{2}=\sigma_{2}^{2}$ *and* $\alpha_{1}=\alpha_{2},\nu_{1}=\nu_{2}$.

**Proof** See Appendix B. ■

Part (A) of Theorem 4 implies that if d≤3, then neither $\sigma^{2}$ nor α are identifiable or consistently estimable, while ν is identifiable. Part (B) implies that when d≥4, all three parameters - $\sigma^{2},\alpha$ and ν-are identifiable. In Euclidean space, the smoothness parameter ν is typically assumed to be known and fixed when discussing fixed-domain asymptotic inference. In this specific Euclidean setting, assuming $\nu_{1}=\nu_{2}=\nu$, (A) still holds while (B) holds for d>4;d=4 is still an unresolved problem in Euclidean space unless the domain is assumed to be bounded (Bolin and Kirchner, 2021). This difference in behaviour between (A) and (B) can be attributed to the integral test being a sufficient condition in Euclidean spaces, which ensures only the equivalence of measures when d≤3; (see Zhang, 2004, for details). In d>4, Anderes (2010) estimated the principal irregular term without the integral test and constructed consistent estimators for α and $\sigma^{2}$ directly. However, this construction does not hold for d=4.

In contrast, the series test in Lemma 3 is a *sufficient and necessary* condition so that we can provide a condition for the equivalence of two measures with Matérn covariograms over any dimension. The dimension also plays an important role in the manifold setting due to Weyl’s Law (Li, 1987; Canzani, 2013). That is, the growth of the eigenvalues and their multiplicities are intertwined with the dimension d; further details are provided within the proof in Appendix B. Another benefit of the sufficient and necessary condition is that the series test can be applied to the squared exponential covariogram, also known as the radial basis function, which can be viewed as a limiting case of the Matérn covariogram when ν→∞, as introduced in Definition 1. Since the spectral density is not a polynomial, the integral test over Euclidean domains is invalid and the conditions for the equivalence of two squared exponential covariograms are intractable. In contrast, the following theorem resolves the equivalence of squared exponential covariograms on a compact manifold ℳ.

**Theorem 5** *Let* $P_{i}$, *for* i=1,2, *be Gaussian measures with squared exponential covariograms parametrised by* $\theta_{i}=\left\{\sigma_{i}^{2},\alpha_{i}\right\}$. *Then* $P_{1}\equiv P_{2}$ *if and only if* $\sigma_{1}^{2}=\sigma_{2}^{2}$ *and* $\alpha_{1}=\alpha_{2}$.

**Proof See** Appendix C. ■

Theorem 5 shows that it is possible to have consistent estimators for both $\sigma^{2}$ and α. So far we have developed formal results on the identifiability of parameters in the covariogram on a compact Riemannian manifold. Inference for identifiable parameters will proceed in customary fashion so we turn our attention to non-identifiable settings, i.e., the Matérn covariogram with known ν on manifolds with dimension d≤3.

### 3.3. Consistency of maximum likelihood estimation

Since ℳ is compact, there is no increasing-domain asymptotic framework and $\cup_{n=1}^{\infty }\left\{x_{n}\right\}$ is always bounded. In the remaining sections, we assume that $\cup_{n=1}^{\infty }\left\{x_{n}\right\}$ is infinite, which is the standard assumption also known as the increasing sequence assumption (also see Stein, 1999; Zhang, 2004; Kaufman and Shaby, 2013). Let $\left\{\sigma_{0},\alpha_{0}\right\}$ be the data generating parameter (oracle) and let ${\hat{\sigma }}_{1,n}^{2}$ be the maximum likelihood estimate of $\sigma^{2}$ obtained by maximising $L_{n}\left(\sigma^{2},\alpha_{1}\right)$ with a misspecified $\alpha_{1}$. The following theorem is analogous to Theorem 3 in Zhang (2004) for compact Riemannian manifolds.

**Theorem 6** *Under the setting of Theorem 4, assuming* $\cup_{n=1}^{\infty }\left\{x_{n}\right\}$ *is infinite, we obtain*

$$\frac{{\hat{\sigma }}_{1,n}^{2}}{C_{\nu ,\alpha_{1}}}\overset{n\to \infty }{⟶}\frac{\sigma_{0}^{2}}{C_{\nu ,\alpha_{0}}},P_{0}\;\text{a.s.}\;$$

**Proof** See Appendix D. ■

In Euclidean space, ${\hat{\sigma }}_{1,n}^{2}/C_{\nu ,\alpha_{1}}$ is asymptotically Gaussian. We conjecture that this asymptotic normality still holds on Riemannian manifolds. However, this result relies on specific constructions in Euclidean space (Wang, 2010), which become invalid for manifolds. A formal proof is beyond the scope of the current manuscript and we intend to pursue this development in future investigations. In Section 4 we present a numerical simulation experiment to demonstrate the asymptotic (normal) behaviour of this parameter on spheres.

### 3.4. Prediction

Given a new location $x_{0}\in ℳ\setminus {\left\{x_{i}\right\}}_{i=1}^{n}$, the best linear unbiased predictor for $Z_{0}=Z\left(x_{0}\right)$ under a covariance function $k_{\rho }$ characterised by its spectral density ρ is given by

$${\hat{Z}}_{n}(\rho )=\gamma_{n}(\rho {)}^{T}\Gamma_{n}(\rho {)}^{-1}Z_{n},$$

where $\gamma_{n}(\rho )=\frac{1}{\sigma^{2}}k_{\rho }\left(x_{0},x_{i}\right)$ and ${\left\{\Gamma_{n}(\rho )\right\}}_{ij}=\frac{1}{\sigma^{2}}k_{\rho }\left(x_{i},x_{j}\right)$.

Kirchner and Bolin (2022) and Bolin and Kirchner (2021) generalise the results of asymptotic optimality of the BLUP based on a misspecified scale parameter in Euclidean spaces (Stein, 1993) to metric spaces. That is, the prediction error of the BLUP under a misspecified scale parameter is asymptotically the same as the error of the BLUP under the true parameter. If the domain is a compact Riemannian manifold and the covariograms are Matérn, then two covariance operators share the same eigenbasis; this is the setting described in Section 5.1 of Kirchner and Bolin (2022) as a special case of Theorem 3.1 therein. We rephrase it in the following lemma with some modifications to fit the Matérn covariograms on a compact Riemannian manifold with a different and simpler proof.

**Lemma 7** *Let* $\rho_{0},\rho_{1}$ *be the spectral densities of two Gaussian measures on* ℳ *with Matérn covariograms. Given* $x_{0}\in ℳ\setminus {\left\{x_{i}\right\}}_{i=1}^{n}$, let ${\hat{Z}}_{n}\left(\rho_{i}\right)$ *be the best linear unbiased predictor of* $Z_{0}:=Z\left(x_{0}\right)$ *based on observations* $\left\{Z\left(x_{1}\right),\cdots ,Z\left(x_{n}\right)\right\}$ *with* ${\left\{x_{i}\right\}}_{i=1}^{\infty }$ *being infinite and having* $x_{0}$ *as an accumulation point, where $\rho_{i}$ is the spectral density of* Z(⋅). If there exists a real number c such that ${lim}_{m\to \infty }\;\frac{\rho_{1}(m)}{\rho_{0}(m)}=c$, *then*:

-
$$\frac{E_{\rho_{0}}{\left({\hat{Z}}_{n}\left(\rho_{1}\right)-Z_{0}\right)}^{2}}{E_{\rho_{0}}{\left({\hat{Z}}_{n}\left(\rho_{0}\right)-Z_{0}\right)}^{2}}\overset{n\to \infty }{⟶}1$$
-
$$\frac{E_{\rho_{1}}{\left({\hat{Z}}_{n}\left(\rho_{1}\right)-Z_{0}\right)}^{2}}{E_{\rho_{0}}{\left({\hat{Z}}_{n}\left(\rho_{1}\right)-Z_{0}\right)}^{2}}\overset{n\to \infty }{⟶}c$$

**Proof** See Appendix E. ■

Focusing on the parameters in a Matérn covariogram, let ${\hat{\sigma }}_{1,n}^{2}$ be the maximum likelihood estimate of $L_{n}\left(\sigma^{2},\alpha_{1}\right)$ and $\rho_{i}$ be the spectral density of the Matérn covariogram with decay parameter $\alpha_{i}$.

**Theorem 8** *Under the same conditions as in Theorem & and Lemma* 7, *let* $\sigma_{1}^{2}=\sigma_{0}^{2}C_{\nu ,\alpha_{1}}/C_{\nu ,\alpha_{0}}$, *then*

$$\frac{E_{\sigma_{0}^{2},\alpha_{0}}{\left({\hat{Z}}_{n}\left(\alpha_{1}\right)-Z_{0}\right)}^{2}}{E_{\sigma_{0}^{2},\alpha_{0}}{\left({\hat{Z}}_{n}\left(\alpha_{0}\right)-Z_{0}\right)}^{2}}\;\overset{n\to \infty }{\to }1,\frac{E_{{\hat{\sigma }}_{1,n}^{2},\alpha_{1}}{\left({\hat{Z}}_{n}\left(\alpha_{1}\right)-Z_{0}\right)}^{2}}{E_{\sigma_{0}^{2},\alpha_{0}}{\left({\hat{Z}}_{n}\left(\alpha_{1}\right)-Z_{0}\right)}^{2}}\overset{n\to \infty }{\underset{P_{0}\text{ a.s.}}{\to }}1.$$

**Proof** See Appendix F. ■

Note that Lemma 7 and Theorem 8 offer the manifold versions of Theorems 3 and 4 in Kaufman and Shaby (2013).

## 4. Matérn on spheres

We now consider Gaussian processes with the Matérn covariogram on the d-dimensional sphere $S^{d}$, including two popular manifolds in spatial statistics: the circle $S^{1}$ and sphere $S^{2}$. We show that all theorems in the previous sections hold for $S^{d}$ with d=1,2,3. As earlier, we assume that $P_{i},i=1,2$, are two Gaussian measures on $S^{d}$ with Matérn covariogram parameters $\left\{\sigma_{i}^{2},\alpha_{i},\nu \right\}$.

**Theorem 9** *For spheres with dimension* d=1,2,3, *the following results are true*:

1. $P_{1}\equiv P_{2}$ *if and only if* $\sigma_{1}^{2}/C_{\nu ,\alpha_{1}}=\sigma_{2}^{2}/C_{\nu ,\alpha_{2}}$, *so neither* $\sigma^{2}$ nor α *can be consistently estimated*.
2. *Let the data generating parameters be* $\left\{\sigma_{0},\alpha_{0}\right\}$ *and* ${\hat{\sigma }}_{1,n}^{2}$ *be the maximum likelihood estimation of* $L_{n}\left(\sigma^{2},\alpha_{1}\right)$ *with misspecified* $\alpha_{1}$ *based on increasing sequence* ${\left\{x_{i}\right\}}_{i=1}^{n}$. *Then*,
$$\frac{{\hat{\sigma }}_{1,n}^{2}}{C_{\nu ,\alpha_{1}}}\overset{n\to \infty }{⟶}\frac{\sigma_{0}^{2}}{C_{\nu ,\alpha_{0}}},P_{0}\;\text{a.s.}\;$$
3. *Given* $x_{0}\in ℳ\setminus {\left\{x_{i}\right\}}_{i=1}^{n}$, *let* ${\hat{Z}}_{n}$ *be the best linear unbiased predictor of* $Z_{0}:=Z\left(x_{0}\right)$ *based on observations* $\left\{Z\left(x_{1}\right),\cdots ,Z\left(x_{n}\right)\right\}$ *with* ${\left\{x_{i}\right\}}_{i=1}^{\infty }$ *being infinite, then*
$$\frac{E_{{\hat{\sigma }}_{1,n}^{2},\alpha_{1}}{\left({\hat{Z}}_{n}\left(\alpha_{1}\right)-Z_{0}\right)}^{2}}{E_{\sigma_{0}^{2},\alpha_{0}}{\left({\hat{Z}}_{n}\left(\alpha_{1}\right)-Z_{0}\right)}^{2}}\overset{n\to \infty }{⟶}1,P_{0}\;\text{a.s.}\;$$

**Proof** See Appendix G. ■

Next, we consider two concrete examples: the circle $S^{1}$ and the sphere $S^{2}$.

### 4.1. Matérn covariogram on circle

First, we recall the simplified form of the Matérn covariogram on $S^{1}$ (Borovitskiy et al., 2020):

**Lemma 10** When $ℳ=S^{1}\subset R^{2}$ and ν=1/2+s,s∈N, *the Matérm covariogram is given by*

(3) $$k\left(x,y\right)=\frac{\sigma^{2}}{C_{\nu ,\alpha }^{\prime }}\overset{s}{\underset{k=0}{\sum }}\;a_{s,k}{\left(\alpha \left(\left|x-y\right|-1/2\right)\right)}^{k}{\text{hyp}}^{k}\left(\alpha \left(\left|x-y\right|-1/2\right)\right),\;x,y\in S^{1},$$

*where* $C_{\nu ,\alpha }^{\prime }$ *is chosen so that* $k(x,x)=\sigma^{2},\;hyp{\;}^{k}$ *is cosh when* k *is even and sinh when* k *is odd*, $a_{s,k}$ *are constants depending on* ν and α; *see* Borovitskiy et al. (2020) *for details*.

Note that $x-y:=\theta_{x}-\theta_{y}\;mod1$ for $x=e^{2\pi i\theta_{x}}$ and $y=e^{2\pi i\theta_{y}}$. Therefore, the Matérn covariogram is “stationary” with respect to this group addition instead of the standard addition in Euclidean space. The corresponding spectral density is given by

(4) $$\rho \left(n\right)=\frac{2\sigma^{2}\alpha \text{sinh}\;\left(\frac{\alpha }{2}\right)}{C_{\nu ,\alpha }^{\prime }(2\pi {)}^{1-2\nu }}{\left(\alpha^{2}+4\pi^{2}n^{2}\right)}^{-\nu -\frac{1}{2}},\;n\in Z.$$

In particular, when ν=1/2, the covariogram and spectral densities admit simple forms:

$$\begin{array}{rr} k(x,y) & \;=\frac{\sigma^{2}}{cosh\;(\alpha /2)}cosh\;(\alpha (|x-y|-1/2)),\\ \rho (n) & \;=2\sigma^{2}\alpha tanh\;(\alpha /2){\left(\alpha^{2}+4\pi^{2}n^{2}\right)}^{-1}. \end{array}$$

**Figure 1:**
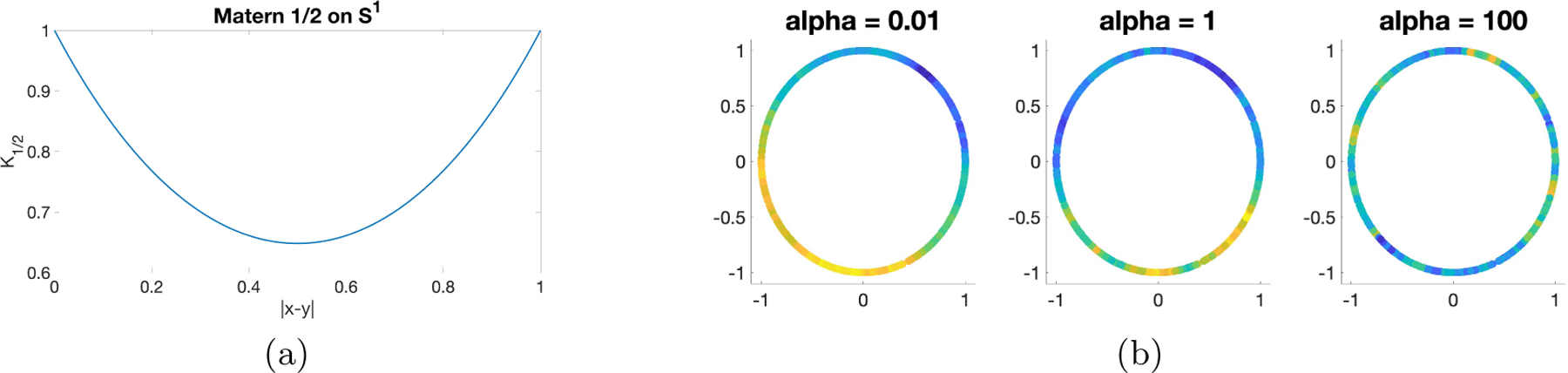
(a) Covariogram of Matérn 1/2 on $S^{1}$; (b): Sample fields with $\sigma^{2}=0.1,\nu =1/2,\;\alpha \in \{0.01,1,100\}$.

Figure 1(a) depicts a covariogram with ν=1/2,α=2, and $\sigma^{2}=1$. Note that |x-y|=1/2 means that x and y are antipodal points so the correlation attains a minimum. Figure 1(b) shows a set of simulated ‘s with different values of α. It is clear that the smaller values of α generate smoother random fields as the correlation grows larger.

**Corollary 11** *Let* ν=1/2, *then* $P_{1}\equiv P_{2}$ *if and only if* $\sigma_{1}^{2}\alpha_{1}\text{tanh}\;\left(\alpha_{1}/2\right)=\sigma_{2}^{2}\alpha_{2}tanh\;\left(\alpha_{2}/2\right)$, *so neither* $\sigma^{2}$ nor α can be consistently estimated.

For a general ν=1/2+s,s∈N, the normalising constant is

$$C_{\nu ,\alpha }^{\prime }=\sum_{k=0}^{s}\;a_{s,k}(-\alpha /2{)}^{k}{hyp}^{k}\;(-\alpha /2).$$

We point out that this $C_{\nu ,\alpha }^{\prime }$ is different from the $C_{\nu ,\alpha }^{\prime }$ in Definition 1 when $ℳ=S^{1}$. Although we cannot express $C_{\nu ,\alpha }^{\prime }$ as an elementary function, we can still find the microergodic parameter for any =s+1/2,s∈Z :

**Corollary 12** *Let* ν=1/2+s,s∈Z, *then* $P_{1}\equiv P_{2}$ *if and only if* $\sigma_{1}^{2}\alpha_{1}\text{sinh}\;\left(\alpha_{1}/2\right)/C_{\nu ,\alpha_{1}}^{\prime }=\sigma_{2}^{2}\alpha_{2}sinh\;\left(\alpha_{2}/2\right)/C_{\nu ,\alpha_{2}}^{\prime }$, *so neither* $\sigma^{2}$ *nor* α *can be consistently estimated*.

**Figure 2:**
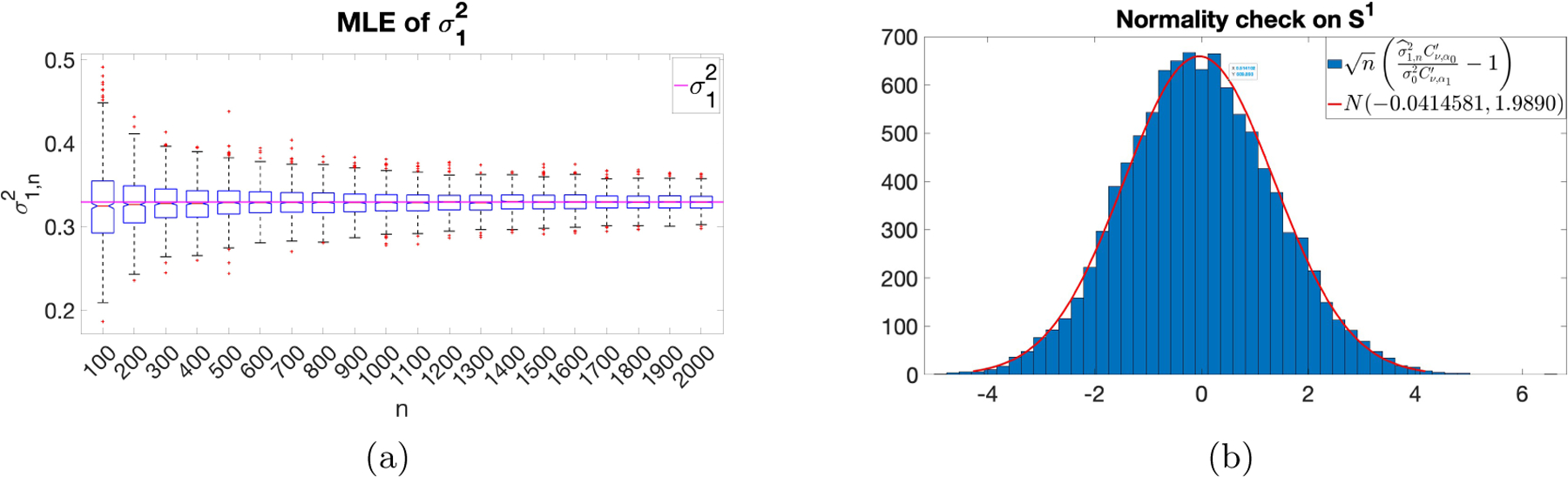
(a) ${\hat{\sigma }}_{1,n}^{2}$ v.s. $\sigma_{1}^{2}$; (b): Distribution of $\sqrt{n}\left(\frac{{\hat{\sigma }}_{1,n}^{2}}{\sigma_{1}^{2}}-1\right)$.

Figure 2 shows that ${\hat{\sigma }}_{1,n}^{2}\to \sigma_{1}^{2}:=\frac{\sigma_{0}^{2}\alpha_{0}sinh\;\left(\alpha_{0}/2\right)}{C_{\nu ,\alpha_{0}}^{\prime }}\frac{C_{\nu ,\alpha_{1}}^{\prime }}{\alpha_{1}sinh\;\left(\alpha_{1}/2\right)}$ as shown by the horizontal line and the empirical distribution of $\sqrt{n}\left(\frac{{\hat{\sigma }}_{1,n}^{2}}{\sigma_{1}^{2}}-1\right)$ is N(0,2), for $\nu =1/2,\sigma_{0}=0.1,\;\alpha_{0}=2\neq \alpha_{1}=1$. Panel (a) supports Theorem 9 empirically. That is, although $\left(\sigma^{2},\alpha ,\nu \right)$ are not consistently estimable, the microergodic parameter $\frac{\sigma^{2}\alpha sinh\;(\alpha /2)}{C_{\nu ,\alpha }^{\prime }}$ is consistently estimable. Panel (b) supports our conjecture after Theorem 6 empirically.

### 4.2. Matérn covariogram on the sphere

On a sphere $S^{2}$, the Mateŕn covariogram is more complicated (Borovitskiy et al., 2020):

**Lemma 13** *The Matérn covariogram on* $ℳ=S^{2}$ *with* ν>0 is

$$k(x,y)=\frac{\sigma^{2}}{C_{\nu ,\alpha }}\sum_{l=0}^{\infty }\;{\left(\alpha^{2}+l(l+1)\right)}^{-\nu -1}c_{l}{ℒ}_{l}\left(cos\;\left(d_{M}(x,y)\right)\right)$$

*and its spectral density is given by*

$$\rho \left(l\right)=\frac{\sigma^{2}}{C_{\nu ,\alpha }}{\left(\alpha^{2}+l\left(l+1\right)\right)}^{-\nu -1},$$

where $d_{M}(\cdot ,\cdot )$ is the geodesic distance on $S^{2},{ℒ}_{l}$ is the Legendre polynomial of degree l:

$${ℒ}_{l}(z)=\sum_{k=0}^{\left\lfloor l/2\right\rfloor }\;\;(-1{)}^{k}\frac{l!\left(l-k-\frac{1}{2}\right)!}{k!(l-2k)!}(2z{)}^{n-2k},\;\;\text{and}\;\;c_{l}=\frac{\left(2l+1)\Gamma (3/2\right)}{2\pi^{3/2}},\;C_{\nu ,\alpha }=\frac{\Gamma (3/2)}{8\pi^{5/2}}\sum_{l=0}^{\infty }\;\;(2l+1){\left(2\nu \alpha^{2}+l(l+1)\right)}^{-\nu -1}$$

**Remark 14** The index l in the above covariance function is different from the index l in Definition 1. In fact, each Legendre polynomial corresponds to multiple spherical harmonics, so the spectral density does not contain the $c_{l}$ constants anymore.

Unlike Lemma 10, where ν is required to be a half-integer, here ν can be any positive number. However, the covariogram now involves an infinite series, which needs to be approximated when x≠y. Approximating a function on $S^{2}$ is known as the “scatter data interpolation problem” (Narcowich et al., 1998) and preserving the positive definiteness is known as the stability problem (Kunis, 2009). For the Matérn covargioram considered in this manuscript, we adopt a natural and simple approximation using the partial sum of an infinite series. The following theorem controls the approximation error and ensures the positive definiteness of the approximated covariogram.

**Theorem 15** *For the partial sum*

$$k^{L}(x,y)=\frac{\sigma^{2}}{C_{\nu ,\alpha }}\sum_{l=0}^{L}\;{\left(\alpha^{2}+l(l+1)\right)}^{-\nu -1}c_{l}{ℒ}_{l}\left(cos\;\left(d_{M}(x,y)\right)\right),$$

the approximation error is controlled by

$$\left|k^{L}(x,y)-k(x,y)\right|\leq \epsilon :=\frac{12\pi \sigma^{2}}{\sum_{l}\;\;\left(2l+1\right){\left(\alpha^{2}+l\left(l+1\right)\right)}^{-\nu -1}}L^{-2\nu }.$$

*Given observations* $x_{1},\cdots ,x_{n}$ *with minimal separation* $q={inf}_{i\neq j}\;d\left(x_{i},x_{j}\right)$, *the approximated covariance matrix ${\left\{k^{L}\left(x_{i},x_{j}\right)\right\}}_{ij}$ is positive definite for any*

$$L>{\left(\frac{12\pi n\sigma^{2}}{\xi_{\rho }(q)\sum_{l}\;\;(2l+1){\left(\alpha^{2}+l(l+1)\right)}^{-\nu -1}}\right)}^{\frac{1}{2\nu }},$$

*where* $\xi_{\rho }(q)$ is a constant depending on the spectral density ρ and minimal separation q; see the proof for more details.

Proof See Appendix H. ■

The above result implies that the computational cost is of order $\epsilon^{-\frac{1}{2\nu }}$ as ϵ→0. Larger values of ν imply smoother random fields that require smaller values of N to approximate the covariogram. In practice, we can first calculate $\xi_{\rho }(q)$, which is computationally practicable because of the closed-form representation (see Appendix H for details), and then choose N.

**Figure 3:**
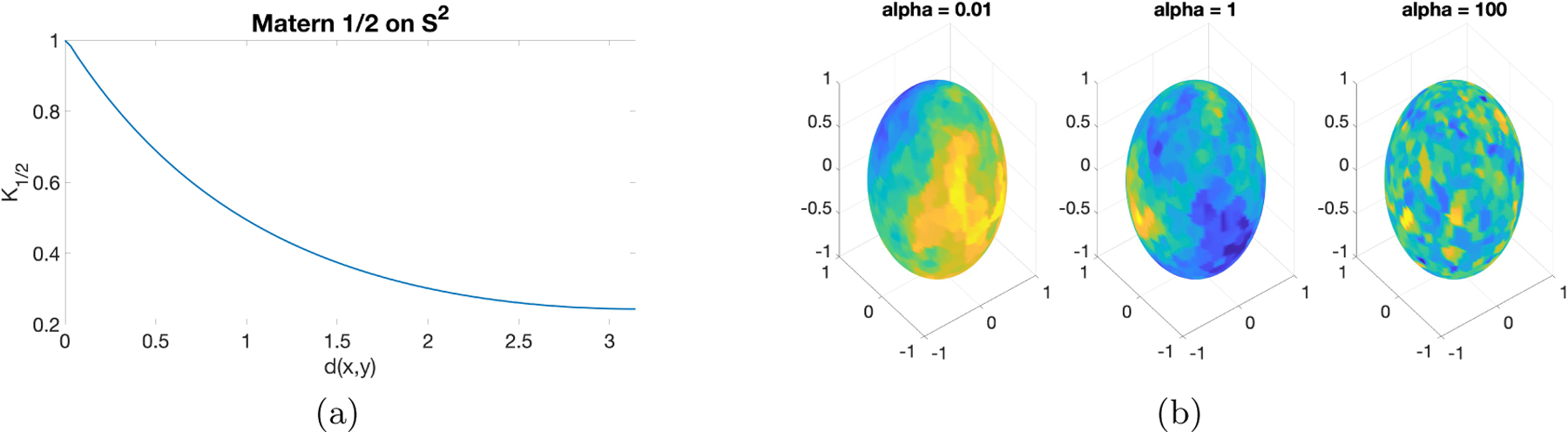
(a) Covariogram of Matérn 1/2 on $S^{2}$; (b): Sample fields with $\sigma^{2}=0.1,\nu =1/2,\;\alpha \in \{0.01,1,100\}$.

Figure 3(a) presents the covariogram with ν=1/2,α=1, and $\sigma^{2}=1$. Note that d(x,y)=π means that x and y are antipodal points so the correlation reaches the minimum. Figure 3(b) shows some simulated Z’s with different α’s. Similar to $ℳ=S^{1}$, smaller values of α lead to smoother random fields.

However, due to the bias introduced by the partial sum, we do not have access to the ground truth covariogram, so the analogue of Figure 2 is not available anymore. Similar issues arise in approximations to the Matérn on a compact manifold (Sanz-Alonso and Yang, 2022a). Instead, we show the theoretical results on microergodic parameters analogous to Corollary 12:

**Corollary 16 $P_{\theta_{1}}\equiv P_{\theta_{2}}$** *if and only if* $\sigma_{1}^{2}/C_{\nu ,\alpha_{1}}=\sigma_{2}^{2}/C_{\nu ,\alpha_{2}}$, *so neither* $\sigma^{2}$ *nor* α *can be consistently estimated*.

## 5. Discussion

This article has formally developed some theoretical results on statistical inference for Gaussian processes with Matérn covariograms on compact Riemannian manifolds. Our focus has primarily been on the identifiability and consistency (or lack thereof) of the covariogram parameters and of spatial predictions. For the Matérn and squared exponential covariograms, we provide a sufficient and necessary condition for the equivalence of two Gaussian random measures through a series test and derive identifiable and consistently estimable microergodic parameters for an arbitrary dimension d. Specifically for d≤3, we formally establish the consistency of maximum likelihood estimates of the parameters and the asymptotic normality of the best linear unbiased predictor under a misspecified decay parameter. The circle and sphere are analysed as two examples with corroborative numerical experiments.

We anticipate that the results developed here will generate substantial future work in this domain. For example, as we have alluded to earlier in the article, in Euclidean spaces we know that the maximum likelihood estimate of $\sigma^{2}$ is asymptotically normal: $\sqrt{n}\left(\frac{{\hat{\sigma }}_{1,n}^{2}}{C_{\nu ,\alpha_{1}}}-\frac{\sigma_{0}^{2}}{C_{\nu ,\alpha_{0}}}\right)\to N(0,2)$. While our numerical experiments lead us to conjecture that an analogous result holds for compact Riemannian manifolds, a formal proof may well require substantial new machinery that we intend to explore further. Next, we conjecture that two measures with the Matérn covariogram are equivalent on $R^{4}$ if and only if they have the same decay and spatial variance parameters. We know this result holds for manifolds with d=4, but a formal proof for $R^{4}$ has not yet been established. Based upon similar reasonings we conjecture that two measures with squared exponential covariograms are equivalent on Euclidean spaces if and only if they have the same decay and spatial variance parameters.

Another future generalisation is to consider covariograms on compact Riemannian manifolds that are not simultaneously diagonalisable, whose asymptotically optimal linear predictor has been studied in Kirchner and Bolin (2022). Nevertheless, issues pertaining to the equivalence of measures, derivation of microergodic parameters and consistency of maximum likelihood estimates remain unresolved. Furthermore, covariograms that offer scientific interpretation in practical inference need to be explored. In this regard, it is worth remarking that although our results are primarily concerned with maximum likelihood estimates, they will provide useful insights into Bayesian learning on manifolds. For example, the failure to consistently estimate certain (non-microergodic) parameters will inform Bayesian modellers that inference for such parameters will always be sensitive to their prior specifications. This will open up new avenues of research in specifying prior distributions for microergodic parameters. Formal investigations into the consistency of the posterior distributions of Matérn covariogram parameters on manifolds are of inferential interest and may benefit from some of our developments in the current manuscript.

Other avenues for future developments will relate to computational efficiency of Gaussian processes on manifolds. Here, a natural candidate for explorations is the tapered covariogram on manifold to introduce sparsity in the covariance matrix (Furrer et al., 2006). Since our domain in the current manuscript is compact, unlike in Euclidean domains, further compact truncation is redundant. One can explore the development of new “tapered” covariograms that achieve positive-definiteness and sparsity. Other approaches that induce dimension reduction based on conditional expectations, such as Gaussian predictive processes (Banerjee et al., 2008), may be explored on compact Riemannian manifolds since these low-dimensional processes are induced by any valid probability measure, although the choice of inputs to define the lower dimensional subspace will need to be addressed. On the other hand, sparse processes resulting from approximations using directed acyclic graphs (Datta et al., 2016b) are less natural for modelling data on manifolds since they depend on well-defined neighbours of inputs, which are less obvious to define outside of Euclidean spaces. Nevertheless, Datta et al. (2016a) developed adaptive Nearest-Neighbour Gaussian processes for massive space-time data sets on Euclidean spaces that selected neighbours using the covariance kernel as a metric for proximity. Such an approach holds promise in modelling massive data sets on manifolds.

In addition, asymptotic properties of estimates under tapering are of interest and have, hitherto, been explored only in Euclidean domains (Kaufman et al., 2008; Du et al., 2009) and without the presence of measurement error processes (“nuggets”). Inference for Gaussian process models with measurement errors (nuggets) on compact manifolds also present novel challenges and can constitute future work. Identifiability and consistency of the nugget in Euclidean spaces have only recently started receiving attention (Tang et al., 2021). However, the developments for Euclidean spaces do not easily apply to compact Riemannian manifolds; hence new tools will need to be developed. On complex or unknown domains, the eigenvalues and eigenfunctions of the Laplacian operator need to be estimated (Belkin and Niyogi, 2007). Asymptotic analysis of estimation in the spectral domain should be closely related to the frequency domain. Finally, since compact manifolds are distinct from non-compact manifolds, both geometrically and topologically, generalisation to non-compact Riemannian manifolds is of interest, where the spectrum is not discrete. Analytic tools on non-compact manifolds will need to be developed.

## Footnotes

1. This article does not consider the noise process, which introduces additional difficulties that are beyond the scope of the current manusript; see Tang et al. (2021) for related developments in Euclidean space.

2. Solin and Kok (2019) provides an alternative definition based on PDEs with boundary conditions.

## Appendix A. Proof of Lemma 3

Before proving Lemma 3, we recall the following lemma (Proposition B, Chapter III Yadrenko, 1983), also known as the Feldman-Hájek theorem:

**Lemma 17** $P_{1}\equiv P_{2}$ *if and only if*

1. Operator $D=B_{1}^{-1/2}B_{2}B_{1}^{-1/2}-I$ is Hilbert-Schmidt;
2. Eigenvalues of D are strictly greater than −1,

where $B_{i}$ is the correlation operator of $P_{i}$ defined by:

$$\left(B_{i}h\right)(x):=\int_{ℳ}\;\sum_{l=0}^{\infty }\;\rho_{i}(l)f_{l}(x)f_{l}(y)h(y){dV}_{g}(y),h\in L^{2}(ℳ).$$

**Proof of Lemma 3**. By Lemma 17, it suffices to check conditions 1 and 2. Let $\gamma_{n}^{i}$ be the eigenvalue of $B_{i}$ and $d_{n}$ be the eigenvalue of D. Observe that $f_{n}$ is an eigenfunction of $B_{i}$ with eigenvalue $\rho_{i}(n)$ :

$$\left(B_{i}f_{l}\right)(x)\;=\int_{ℳ}\;\;\sum_{m}\;\;\rho_{i}(m)f_{m}(x)f_{m}(y)f_{l}(y)dVg(y)\;\;=\sum_{m}\;\;\rho_{i}(m)f_{m}(x)\int_{M}\;\;f_{m}(y)f_{l}(y){dV}_{g}(y)\;\;=\sum_{m}\;\;\rho_{i}(m)f_{m}(x){\left\langle f_{m},f_{l}\right\rangle }_{ℳ}\;\;=\sum_{m}\;\;\rho_{i}(m)f_{m}(x)\delta_{nm}\;\;=\rho_{i}\left(l\right)f_{l}\left(x\right),$$

where δ is the Kronecker delta and $\langle \cdot ,\cdot \rangle_{ℳ}$ is the $L^{2}$ inner product on ℳ with $f_{n}$ being orthonormal basis.

Since $B_{i}$’s share the same eigenfunctions and hence commute, we have $d_{l}=\frac{\gamma_{l}^{2}}{\gamma_{l}^{1}}-1=\frac{\rho_{2}(l)}{\rho_{1}(l)}-1>-1$, so condition 2 holds by the definition of $\rho_{i}$. For condition 1, observe that $d_{l}=\frac{\rho_{2}(l)-\rho_{1}(l)}{\rho_{1}(l)}$, so

$$D\;\text{is Hilbert-Schmidt}\;⟺\sum_{l}\;d_{l}^{2}<\infty ⟺\sum_{l}\;{\left|\frac{\rho_{2}(l)-\rho_{1}(l)}{\rho_{1}(l)}\right|}^{2}<\infty .$$

■

## Appendix B. Proof of Theorem 4

**Proof** We start with (A). First assume that $\nu_{1}=\nu_{2}=\nu$ and $\sigma_{1}^{2}/C_{\nu ,\alpha_{1}}=\sigma_{2}^{2}/C_{\nu ,\alpha_{2}}$, then observe

$$\left|\frac{\rho_{2}(l)-\rho_{1}(l)}{\rho_{1}(l)}\right|\;=\left|\frac{{\left(\alpha_{1}^{2}+\lambda_{l}\right)}^{\nu +d/2}}{{\left(\alpha_{2}^{2}+\lambda_{l}\right)}^{\nu +d/2}}-1\right|\;\;\leq \left|{\left(\alpha_{1}^{2}+\lambda_{l}\right)}^{\nu +d/2}-{\left(\alpha_{2}^{2}+\lambda_{l}\right)}^{\nu +d/2}\right|/\lambda_{l}^{\nu +d/2}\;\;\leq |\left({\left(\alpha_{1}^{2}/\lambda_{l}+1\right)}^{\nu +d/2}-\left({\left(\alpha_{2}^{2}/\lambda_{l}+1\right)}^{\nu +d/2}|.\right.\right.$$

Note that $(1/x+1{)}^{a}=1+a/x+O\left(x^{-2}\right)$ as x→∞, then when l is sufficiently large so that $\lambda_{l}>0$,

$$\left|{\left(\alpha_{1}^{2}/\lambda_{l}+1\right)}^{\nu +d/2}-{\left(\alpha_{2}^{2}/\lambda_{l}+1\right)}^{\nu +d/2}\right|\leq (\nu +d/2)\left(\alpha_{1}^{2}-\alpha_{2}^{2}\right)\lambda_{l}^{-1}+O\left(\lambda_{l}^{-2}\right)=O\left(\lambda_{l}^{-1}\right).$$

As a result,

$$\sum_{l}\;{\left|\frac{\rho_{2}(l)-\rho_{1}(l)}{\rho_{1}(l)}\right|}^{2}≲\sum_{l}\;\lambda_{l}^{-2}.$$

By Weyl’s law (equation (4.1) in Grebenkov and Nguyen (2013)), $\lambda_{l}\sim l^{2/d}$, so we have $\lambda_{l}^{-2}\sim l^{-d/4}$ hence -4/d<-1 when d≤3. By the series test in Lemma $3,P_{1}\equiv P_{2}$.

For the other direction, observe that

$$\left|\frac{\rho_{2}(l)}{\rho_{1}(l)}\right|=\left|\frac{\sigma_{2}^{2}C_{\nu_{1},\alpha_{1}}{\left(\alpha_{1}^{2}+\lambda_{l}\right)}^{\nu_{1}+d/2}}{\sigma_{1}^{2}C_{\nu_{2},\alpha_{2}}{\left(\alpha_{2}^{2}+\lambda_{l}\right)}^{\nu_{2}+d/2}}\right|\to \left\{\begin{array}{ll} \infty & \nu_{1}<\nu_{2}\\ 1 & \nu_{1}=\nu_{2}\\ 0 & \nu_{1}>\nu_{2}. \end{array}.\right.$$

As a result, if $\nu_{1}\neq \nu_{2},\sum_{l}\;\left|\frac{\rho_{2}(l)}{\rho_{1}(l)}-1\right|\to \infty$ so $P_{1}≢P_{2}$ by the series test.

Then assume $\nu_{1}=\nu_{2}=\nu$ and $\sigma_{1}^{2}/C_{\nu ,\alpha_{1}}\neq \sigma_{2}^{2}/C_{\nu ,\alpha_{2}}$. Let $\sigma_{0}^{2}=\sigma_{2}^{2}\frac{C_{\nu ,\alpha_{1}}}{C_{\nu ,\alpha_{2}}}\neq \sigma_{1}^{2}$, then

$$\sigma_{0}^{2}/C_{\nu ,\alpha_{1}}=\sigma_{2}^{2}/C_{\nu ,\alpha_{2}},$$

so $k\left(\cdot ;\sigma_{0}^{2},\alpha_{1}\right)$ and $k\left(\cdot ;\sigma_{2}^{2},\alpha_{2}\right)$ define two equivalent measures, denoted by $P_{0}$ and $P_{2}$. Observe that

$$k\left(x,y;\sigma_{1}^{2},\alpha_{1}\right)=\frac{\sigma_{1}^{2}}{\sigma_{0}^{2}}k\left(x,y;\sigma_{0}^{2},\alpha_{1}\right)$$

then the corresponding spectral densities $\rho_{0}$ and $\rho_{1}$ only differ by a multiplicative scalar $\frac{\sigma_{1}^{2}}{\sigma_{0}^{2}}$ so $\sum_{l}\;{\left|\frac{\rho_{1}(l)-\rho_{0}(l)}{\rho_{1}(l)}\right|}^{2}=\sum_{l}\;{\left|\frac{\sigma_{1}^{2}-\sigma_{0}^{2}}{\sigma_{1}^{2}}\right|}^{2}=\infty$. So by Lemma $3,P_{0}$ is orthogonal to $P_{1}$, so is $P_{2}$, which is equivalent to $P_{0}$. Now we conclude that $P_{1}\equiv P_{2}$ if and only if $\sigma_{1}^{2}/C_{\nu ,\alpha_{1}}=\sigma_{2}^{2}/C_{\nu ,\alpha_{2}}$ and $\nu_{1}=\nu_{2}$.

Then we show (B). As proved in (A), $P_{1}≢P_{2}$ if $\nu_{1}\neq \nu_{2}$ so we assume $\nu_{1}=\nu_{2}=\nu$. Recall that $\lambda_{n}\to \infty$, so when n is sufficiently large, $\lambda_{n}>\alpha^{2}$, then

(5)
$$\left|\frac{\rho_{2}(l)-\rho_{1}(l)}{\rho_{1}(l)}\right|=\left|\frac{\sigma_{2}^{2}C_{\nu ,\alpha_{1}}{\left(\alpha_{1}^{2}+\lambda_{l}\right)}^{\nu +d/2}}{\sigma_{1}^{2}C_{\nu ,\alpha_{2}}{\left(\alpha_{2}^{2}+\lambda_{l}\right)}^{\nu +d/2}}-1\right|\;\;\geq \frac{\left|\sigma_{2}^{2}C_{\nu ,\alpha_{1}}{\left(\alpha_{1}^{2}+\lambda_{l}\right)}^{\nu +d/2}-\sigma_{1}^{2}C_{\nu ,\alpha_{2}}{\left(\alpha_{2}^{2}+\lambda_{l}\right)}^{\nu +d/2}\right|}{\sigma_{1}^{2}C_{\nu ,\alpha_{2}}{\left(2\lambda_{l}\right)}^{\nu +d/2}}\;\;=2^{-\nu -d/2}\left|\frac{\sigma_{2}^{2}C_{\nu ,\alpha_{1}}}{\sigma_{1}^{2}C_{\nu ,\alpha_{2}}}{\left(\alpha_{1}^{2}/\lambda_{l}+1\right)}^{\nu +d/2}-{\left(\alpha_{2}^{2}/\lambda_{l}+1\right)}^{\nu +d/2}\right|\;\;=2^{-\nu -d/2}\left|\frac{\sigma_{2}^{2}C_{\nu ,\alpha_{1}}}{\sigma_{1}^{2}C_{\nu ,\alpha_{2}}}-1+\left(\nu +\frac{d}{2}\right)\left(\frac{\sigma_{2}^{2}C_{\nu ,\alpha_{1}}}{\sigma_{1}^{2}C_{\nu ,\alpha_{2}}}\alpha_{1}^{2}-\alpha_{2}^{2}\right)\lambda_{l}^{-1}+O\left(\lambda_{l}^{-2}\right)\right|.$$

When $\sigma_{1}^{2}\neq \sigma_{2}^{2}$ or $\alpha_{1}\neq \alpha_{2}$, the constant term $\frac{\sigma_{2}^{2}C_{\nu ,\alpha_{1}}}{\sigma_{1}^{2}C_{\nu ,\alpha_{2}}}-1$ and the linear coefficient $\frac{\sigma_{2}^{2}C_{\nu ,\alpha_{1}}}{\sigma_{1}^{2}C_{\nu ,\alpha_{2}}}\alpha_{1}^{2}-\alpha_{2}^{2}$ in Equation (5) do not vanish at the same time hence $\left|\frac{\rho_{2}(l)-\rho_{1}(l)}{\rho_{1}(l)}\right|≳\lambda_{l}^{-1}$. Then

$$\sum_{l}\;{\left|\frac{\rho_{2}(l)-\rho_{1}(l)}{\rho_{1}(l)}\right|}^{2}≳\sum_{l}\;\lambda_{l}^{-2}=\sum_{l}\;l^{-4/d}=\infty$$

since d≥4. By the series test, $P_{1}≢P_{2}$. When $\sigma_{1}^{2}=\sigma_{2}^{2}$ and $\alpha_{1}=\alpha_{2},P_{1}=P_{2}$ so $P_{1}\equiv P_{2}$, which finises the proof of (B). ■

## Appendix C. Proof of Theorem 5

Proof First assume $\alpha_{1}\neq \alpha_{2}$, or $\alpha_{1}<\alpha_{2}$ without loss of generality, then

$$\left|\frac{\rho_{2}(l)-\rho_{1}(l)}{\rho_{1}(l)}\right|=\left|\frac{\sigma_{2}^{2}C_{\alpha_{1}}}{\sigma_{1}^{2}C_{\alpha_{2}}}e^{-\frac{\lambda_{l}}{2}\left(\frac{1}{\alpha_{2}^{2}}-\frac{1}{\alpha_{1}^{2}}\right)}-1\right|\to \infty$$

since $\lambda_{l}\to \infty$. As a result,

$$\sum_{l}\;{\left|\frac{\rho_{2}(l)-\rho_{1}(l)}{\rho_{1}(l)}\right|}^{2}=\infty .$$

Then assume $\alpha_{1}=\alpha_{2}$ but $\sigma_{1}^{2}\neq \sigma_{2}^{2}$, similarly,

$$\sum_{l}\;{\left|\frac{\rho_{2}(l)-\rho_{1}(l)}{\rho_{1}(l)}\right|}^{2}=\sum_{l}\;{\left(\frac{\sigma_{2}^{2}}{\sigma_{1}^{2}}-1\right)}^{2}=\infty .$$

Then the series test applies. ■

## Appendix D. Proof of Theorem 6

**Proof** Let $\sigma_{1}^{2}=\frac{\sigma_{0}^{2}C_{\nu ,\alpha_{1}}}{C_{\nu ,\alpha_{0}}}$ so $P_{0}\equiv P_{1}$ by Theorem 4. It suffices to show ${\hat{\sigma }}_{1,n}^{2}\to \sigma_{1}^{2},P_{1}$ a.s. Recall that ${\hat{\sigma }}_{1,n}^{2}=\frac{Z_{n}^{T}\Gamma_{n}^{-1}\left(\alpha_{1}\right)Z_{n}}{n}$ and $Z_{n}\sim N\left(0,\sigma_{1}^{2}\Gamma_{n}\left(\alpha_{1}\right)\right)$ under $P_{1}$, where ${\left(\Gamma_{n}(\alpha )\right)}_{i,j}=$ $\frac{1}{C_{\nu ,\alpha }}\sum_{l=0}^{\infty }\;{\left(2\nu \alpha^{2}+\lambda_{l}\right)}^{-\nu -\frac{d}{2}}f_{l}\left(x_{i}\right)f_{l}\left(x_{j}\right)$. As a result, ${\hat{\sigma }}_{1,n}^{2}=\sigma_{1}^{2}\frac{\chi_{n}^{2}}{n}\to \sigma_{1}^{2},P_{1}$ a.s., as n→∞. ■

## Appendix E. Proof of Lemma 7

**Proof** The logic of the proof is similar to the proof of Theorem 1 and 2 in Stein (1993). However, these two theorems are not directly applicable due to the discreteness of spectrum in our case. To be more specific, the key construction in the proof of Stein (1993) is the following. By the assumption, for any ε>0, there exists $M_{\varepsilon }>0$ such that ${sup}_{m\geq M_{\varepsilon }}\;\left|\frac{\rho_{1}(m)}{c\rho_{0}(m)}-1\right|<\varepsilon$. We define

$$\eta_{\varepsilon }\left(m\right)≔\left\{\begin{array}{ll} \frac{1}{c}\rho_{1}\left(m\right) & m\leq M_{\varepsilon }\\ \rho_{0}\left(m\right) & m>M_{\varepsilon } \end{array}\right..$$

That is, $\eta_{\varepsilon }$ differs from $\rho_{0}$ only on a bounded subset of N. Note that in Stein (1993), the key step is to show $P_{\eta_{\varepsilon }}\equiv P_{\rho_{0}}$, and the rest of the proof will not rely on any special structure of the Euclidean domain anymore. That is, it suffices to show $P_{\eta_{\varepsilon }}\equiv P_{\rho_{0}}$, which is a direct consequence of the series test in Lemma 3. The rest of the proof of (i) naturally follows the proof of Theorem 1 in Stein (1993) while the proof of (ii) follows the proof of Theorem 2 in Stein (1993), where $e\left(x_{0},n,f_{1}\right)$ in Stein (1993) corresponds to $Z_{0}-{\hat{Z}}_{n}\left(\rho_{1}\right)$ in our paper. ■

## Appendix F. Proof of Theorem 8

**Proof** For $\sigma_{1}^{2}=\frac{\sigma_{0}^{2}C_{\nu ,\alpha_{1}}}{C_{\nu ,\alpha_{0}}}$, let $\rho_{1}$ and $\rho_{0}$ be the spectral density of the Gaussian process parametrised by $\left(\alpha_{1},\sigma_{1}^{2}\right)$ and $\left(\alpha_{0},\sigma_{0}^{2}\right)$ hence $\rho_{1}/\rho_{0}\to 1$. Then by (ii) in Lemma 7,

$$\frac{E_{\sigma_{1}^{2},\alpha_{1}}{\left({\hat{Z}}_{n}\left(\alpha_{1}\right)-Z_{0}\right)}^{2}}{E_{\sigma_{0}^{2},\alpha_{0}}{\left({\hat{Z}}_{n}\left(\alpha_{1}\right)-Z_{0}\right)}^{2}}\to 1.$$

Observe that

(6)
$$\frac{E_{{\hat{\sigma }}_{1,n}^{2},\alpha_{1}}{\left({\hat{Z}}_{n}\left(\alpha_{1}\right)-Z_{0}\right)}^{2}}{E_{\sigma_{0}^{2},\alpha_{0}}{\left({\hat{Z}}_{n}\left(\alpha_{1}\right)-Z_{0}\right)}^{2}}=\frac{E_{{\hat{\sigma }}_{1,n}^{2},\alpha_{1}}{\left({\hat{Z}}_{n}\left(\alpha_{1}\right)-Z_{0}\right)}^{2}}{E_{\sigma_{1}^{2},\alpha_{1}}{\left({\hat{Z}}_{n}\left(\alpha_{1}\right)-Z_{0}\right)}^{2}}\frac{E_{\sigma_{1}^{2},\alpha_{1}}{\left({\hat{Z}}_{n}\left(\alpha_{1}\right)-Z_{0}\right)}^{2}}{E_{\sigma_{0}^{2},\alpha_{0}}{\left({\hat{Z}}_{n}\left(\alpha_{1}\right)-Z_{0}\right)}^{2}}.$$

The second term in Equation (6) tends to 1. For the first term, by the definition of ${\hat{Z}}_{n}$, we obtain

$$E_{{\hat{\sigma }}_{1,n}^{2},\alpha_{1}}{\left({\hat{Z}}_{n}\left(\alpha_{1}\right)-Z_{0}\right)}^{2}={\hat{\sigma }}_{1,n}^{2}\left(1-\gamma_{n}{\left(\alpha_{1}\right)}^{T}\Gamma_{n}{\left(\alpha_{1}\right)}^{-1}\gamma_{n}\left(\alpha_{1}\right)\right).$$

Hence, the first term in Equation (6) is $\frac{{\hat{\sigma }}_{1,n}^{2}}{\sigma_{1}^{2}}$. Similar to the proof of Theorem $6,{\hat{\sigma }}_{1,n}^{2}=$ $\sigma_{1}^{2}\frac{\chi_{n}^{2}}{n}\to \sigma_{1}^{2},P_{1}:=P_{\sigma_{1}^{2},\alpha_{1}}$ a.s. By Theorem 4, $P_{0}\equiv P_{1}$, so the left hand side of Equation (6) tends to 1, $P_{0}$ a.s. ■

## Appendix G. Proof of Theorem 9

**Proof** d-dimensional spheres are compact Riemannian manifolds. The eigenfunctions of the Laplace operator on $S^{d}$ are known as spherical harmonics, denoted by $S_{m}^{l},m=0,1,\cdots ,\;l=1,\cdots ,t_{d}(m)$. The corresponding eigenvalues are $l(l+d-1)=O\left(l^{2}\right)$ with multiplicity (Müller, 1966; Efthimiou and Frye, 2014)

$$\frac{2l+d-1}{l}\left(\begin{array}{c} l+d-2\\ l-1 \end{array}\right)=O\left(l^{d-1}\right).$$

So 1, 2, 3 follow directly from Theorem 4,6 and 8 respectively. ■

## Appendix H. Proof of Theorem 15

**Proof** First we reformulate the covariogram as

$$k(x,y)=\frac{\sigma^{2}}{C_{\nu ,\alpha }}\sum_{l=0}^{\infty }\;{\left(\alpha^{2}+l(l+1)\right)}^{-\nu -1}c_{l}{ℒ}_{l}\left(cos\;\left(d_{M}(x,y)\right)\right)=C\sum_{l=0}^{\infty }\;a_{l}(z),$$

where $C=\frac{\Gamma (3/2)\sigma^{2}}{2\pi^{3/2}C_{\nu ,\alpha }}=\frac{4\pi \sigma^{2}}{\sum_{l}\;(2l+1){\left(\alpha^{2}+l(l+1)\right)}^{-\nu -1}},z=cos\;\left(d_{M}(x,y)\right)$ and

$$a_{l}(z)={\left(\alpha^{2}+l(l+1)\right)}^{-\nu -1}(2l+1){ℒ}_{l}(z).$$

Observe that ${ℒ}_{l}(z)\in [-1,\;1]$. Therefore,

$$\left|a_{l}(z)\right|\leq {\left(\alpha^{2}+l(l+1)\right)}^{-\nu -1}(2l+1).$$

As a result,

$$\left|k^{L}(x,y)-k(x,y)\right|\;\leq C\sum_{l=L+1}^{\infty }\;\;\left|a_{l}(z)\right|\leq C\sum_{l=L+1}^{\infty }\;\;{\left(\alpha^{2}+l(l+1)\right)}^{-\nu -1}(2l+1)\;\;\leq C\sum_{l=L+1}^{\infty }\;\;{\left(l^{2}\right)}^{-\nu -1}(3l)=3C\sum_{l=L+1}^{\infty }\;\;l^{-2\nu -1}\leq 3C\int_{L+1}^{\infty }\;\;t^{-2\nu -1}\;dt\;\;\leq \frac{3C}{2\nu }L^{-2\nu }=\frac{6\pi \sigma^{2}}{\nu \sum_{l}\;\;\left(2l+1\right){\left(\alpha^{2}+l\left(l+1\right)\right)}^{-\nu -1}}L^{-2\nu }.$$

That is, if the target approximation error is ϵ, then we can truncate the infinite sum at

$$L=\left\lfloor {\left(\frac{6\pi \sigma^{2}}{\epsilon \nu \sum_{l}\;\;(2l+1){\left(\alpha^{2}+l(l+1)\right)}^{-\nu -1}}\right)}^{\frac{1}{2\nu }}\right\rfloor +1.$$

To prove positive definiteness, we first find the lower bound of the minimal eigenvalue of the covariance matrix $\Sigma :={\left\{k\left(x_{i},x_{j}\right)\right\}}_{ij}$, denoted by $\lambda_{\text{min}\;}$. By Theorem 2.8 (i) in Narcowich et al. (1998),

$$\lambda_{\text{min}}\geq \xi_{\rho }\left(q\right):=\Gamma_{\rho }\left(K\right)\left(1-\frac{\pi^{3}k\left(q\right)}{4q}{\left(\frac{\sin\;\left(\frac{q}{2}\right)}{\frac{q}{2}}\right)}^{K}\right),$$

where

$$k(q)=max\left\{\frac{8\pi^{2}}{q},\frac{25\pi }{2}\right\},K=\underset{m\in N}{arg\;min}\left\{m:1-\frac{\pi^{3}k(q)}{4q}{\left(\frac{sin\;(q/2)}{q/2}\right)}^{m}>0\right\},$$

and $\Gamma_{\rho }(K)$ is determined by the spectral density ρ,K and the B-spline, see Equation (2.41) in Narcowich et al. (1998) for further details (where m=2 in our setting). Let the truncated covariance function be $\Sigma^{L}:={\left\{k^{L}\left(x_{i},x_{j}\right)\right\}}_{ij}$ with minimal eigenvalue $\lambda_{min}^{N}$, then by the first half of the proof,

$$\lambda_{min}^{L}\geq \lambda_{min}-∥\Sigma -\Sigma^{L}∥\geq \xi_{\rho }(q)-n{∥\Sigma -\Sigma^{L}∥}_{max}\geq \xi_{\rho }(q)-\frac{6\pi n\sigma^{2}L^{-2\nu }}{\nu \sum_{l}\;\;(2l+1){\left(\alpha^{2}+l(l+1)\right)}^{-\nu -1}}$$

The second inequality follows from a matrix norm equivalence: $∥A{∥}_{max}\leq ∥A∥\leq n∥A{∥}_{max}$ for any n×n matrix A. The first inequality relies on the fact that ${eig}_{min}\;(A)\geq {eig}_{min}\;(B)-∥A-B∥$ for symmetric matrices A and B. Note that ${eig}_{min}\;(A)={min}_{∥x∥=1}\;x^{⊤}Ax$ and let $x_{0}$ be the eigenvector of A associated with the smallest eigenvalue, that is, $Ax_{0}={eig}_{\text{min}\;}\;(A)x_{0}$. By the same observation, $x_{0}^{⊤}Bx_{0}\geq {eig}_{min}\;(B)$. Then,

$${eig}_{min}\;(A)=x_{0}^{⊤}Ax_{0}=x_{0}^{⊤}(B+A-B)x_{0}=x_{0}^{⊤}Bx_{0}+x_{0}^{⊤}(A-B)x_{0}\geq {eig}_{min}\;(B)+x_{0}^{⊤}(A-B)x_{0}.$$

For the last term, since $∥A-B∥={max}_{∥x∥=1}\;\left|x^{⊤}(A-B)x\right|$, we have $\left|x_{0}^{⊤}(A-B)x_{0}\right|\leq ∥A-B∥$, hence $x_{0}^{⊤}(A-B)x_{0}\geq -∥A-B∥$ as desired. Let $\xi_{\rho }(q)-\frac{6\pi n\sigma^{2}L^{-2\nu }}{\nu \sum_{l}\;(2l+1){\left(\alpha^{2}+l(l+1)\right)}^{-\nu -1}}>0$, we have

$$L>{\left(\frac{6\pi n\sigma^{2}}{\nu \xi_{\rho }\left(q\right)\sum_{l}\;\;\left(2l+1\right){\left(\alpha^{2}+l\left(l+1\right)\right)}^{-\nu -1}}\right)}^{\frac{1}{2\nu }}$$
